# Clinical outcome and laboratory markers for predicting disease activity in patients with disseminated opportunistic infections associated with anti-interferon-γ autoantibodies

**DOI:** 10.1371/journal.pone.0215581

**Published:** 2019-04-25

**Authors:** Nasikarn Angkasekwinai, Yupin Suputtamongkol, Pakpoom Phoompoung, Manop Pithukpakorn, Ekkarat Wongswat, Pinklow Umrod, Sasima Tongsai, Suporn Foongladda

**Affiliations:** 1 Department of Medicine, Faculty of Medicine Siriraj Hospital, Mahidol University, Bangkok, Thailand; 2 Department of Immunology, Faculty of Medicine Siriraj Hospital, Mahidol University, Bangkok, Thailand; 3 Department of Microbiology, Faculty of Medicine Siriraj Hospital, Mahidol University, Bangkok, Thailand; Rutgers New Jersey Medical School, UNITED STATES

## Abstract

**Background:**

Clinical courses and treatment outcomes are largely unknown in patients with adult-onset immunodeficiency associated with anti-interferon-gamma autoantibodies due to the fact that it was recently recognized and anti-IFN-γ auto-Abs detection is not widely available.

**Methods and findings:**

Non-HIV-infected adult patients with detectable anti-IFN-γ auto-Abs diagnosed and followed at Siriraj Hospital, Bangkok, Thailand during January 2013 to November 2016 were prospectively studied. At each follow-up visit, patients were classified as stable or active disease according to symptoms and signs, and all proven OIs were recorded. Laboratory parameters, including erythrocyte sedimentation rate, C-reactive protein, and anti-IFN-γ auto-Abs level, were compared between active and stable disease episodes. We identified 80 patients with this clinical syndrome and followed them up during study period. Seventy-nine patients developed overall 194 proven opportunistic infections. *Mycobacterium abscessus* (34.5%) and *Salmonella* spp. (23.2%) were the two most common pathogens identified among these patients. Sixty-three patients were followed for a median of 2.7 years (range 0.6–4.8 years). Eleven (17.5%) patients achieved the drug-free remission period for at least 9 months. Four patients died. Anti-IFN-γ auto-Abs concentration was significantly lower at baseline and decreased over time in the drug-free remission group compared to another group (*p* = 0.001). C-reactive protein, erythrocyte sedimentation rate and white cell count were found to be useful biomarkers for determining disease activity during follow-up.

**Conclusions:**

Reinfection or relapse of OIs is common despite long-term antimicrobial treatment in patients with anti-IFN-γ auto-Abs. Treatment to modify anti-IFN-γ auto-Abs production may improve long-term outcomes in this patient population.

## Introduction

Adult-onset immunodeficiency due to anti-interferon-gamma autoantibodies (anti-IFN-γ auto-Abs) is a distinct clinical syndrome of disseminated nontuberculous mycobacteria (NTM) or other opportunistic (OI) infections in previously healthy adult patients in association with anti-IFN- γ auto-Abs [1]. Since first described in 2004, this syndrome has emerged as a very difficult-to-treat infectious disease in otherwise immunocompetent adults from Asian countries, including Thailand [2–9]. The dysfunction of IFN-γ by naturally occurring neutralizing antibodies against IFN-γ causes disrupted downstream response ability and impaired killing ability capacity intracellular organisms by macrophages[10]. Additional abnormal immune responses in adult-onset immunodeficiency characterized by reduced production of interleukin (IL)-2 and tumor necrosis factor (TNF)-α may be a consequence of T cell exhaustion following chronic antigenic stimulation [11]. As a result, patients with anti-IFN-γ auto-Abs typically present with disseminated infection caused by opportunistic intracellular pathogens, especially NTM [1, 12–14]. A significant proportion of patients are co-infected or present with other intracellular pathogens, including nontyphoidal *Salmonella* spp., *Cryptococcus neoformans*, *Talaromyces marneffei*, and Varicella zoster virus (VZV) [1, 12].

Among many case reports and case series published to date, there are only two case series from Taiwan and Japan describing the clinical features and outcomes of patients with NTM diseases and detectable anti-IFN-γ auto-Abs [12, 13]. Therefore, more information on the clinical courses, outcomes and factors associated with this clinical syndrome, especially in patients with anti-IFN-γ auto-Abs associated with other OIs, is needed. In addition, the value of common laboratory markers, such as C-reactive protein (CRP) or the erythrocyte sedimentation rate (ESR), as an adjunct to clinical assessment of disease activity in the long-term management of these patients is unknown.

## Materials and methods

### Study design and population

From January 2013 to November 2016, we conducted a prospective clinical study of adult patients (aged 18 years or older) in whom anti-IFN-γ auto-Abs levels were detected and we followed them at the Infectious Diseases and Tropical Medicine Clinic, Siriraj Hospital, Bangkok, Thailand. The study protocol was approved by the Siriraj Institutional Review Board (SIRB) (COA no. 070/2556), and a written informed consent was obtained from all study participants.

### Study procedures

The baseline characteristics of our patients were defined as demographic data, clinical characteristics, and laboratory results recorded at the time point when their anti-IFN-γ auto-Abs level was first measured. Medical records were retrospectively reviewed to obtain these data for patients who were diagnosed prior to January 2013. Patients with detectable anti-IFN-γ auto-Abs were asked to return for follow-up visits and for the maintenance antimicrobial treatment every 1–4 months. Patient management, including choice and duration of antimicrobial therapy, was determined by the attending physician based on the pathogens identified, site of infection, and patient tolerance.

Characteristics of infections and the presence of reactive skin diseases associated with this syndrome were recorded. Reactive skin diseases were classified as Sweet syndrome or neutrophilic dermatosis, acute generalized exanthematous pustulosis (AGEP), pustular psoriasis, erythema nodosum (EN), or others, as previously described [14]. Based only on the clinical findings and signs of OIs, the clinical stage of a patient was classified at each visit as stable disease (defined as no fever or symptoms and no signs of active infection) or active disease (new onset of fever or new symptoms and signs, and/or persistent or re-enlargement of preexisting lymph nodes, and/or persistent or reappearance of reactive dermatosis). Baseline laboratory investigations included the following: complete blood count (CBC); blood chemistry, including aspartate transaminase (AST), alanine transaminase (ALT), albumin, globulin, plasma urea, and creatinine; antinuclear antibody (ANA) levels; inflammatory biomarker levels, including CRP and ESR; and immunoglobulin G (IgG), CD4, and CD8, and human leukocyte antigen (HLA) levels. At each episode of an OI, clinical specimens, such as blood culture, lymph node aspiration and/or biopsy, were obtained for causative pathogen identification prior to treatment. At each follow-up visit, CBC, CRP, ESR, and anti-IFN-γ auto-Abs levels were assessed. Other laboratory investigations were performed as clinically indicated.

The overall clinical outcomes of each patient at the end of the study period (30 November 2016) were categorized into two groups: the drug-free remission group (those with stable disease without antimicrobial treatment for at least 9 months) and the non-remission group (those who required either continuation of oral antibiotic treatment, with or without intermittent intravenous antibiotic therapy).

All data were recorded on standardized case record forms. Anti-IFN-γ auto-Abs concentrations in serum were measured using enzyme-linked immunosorbent assay (ELISA), same method as in our previous publications [14, 15]. Briefly, serum was 100-fold diluted with phosphate-buffered saline-0.1% Tween®20 (PBST) containing 1% bovine serum albumin (BSA). A volume of 50 μL of diluted sera was transferred to a 96-well ELISA plate (Corning Inc., Corning, NY) coated with 0.5 μg/ml of recombinant human IFN-γ (R&D Systems, Minneapolis, MA) and incubated at room temperature for one hour. After washing, IFN-γ autoantibodies were detected by alkaline phosphatase-conjugated goat anti-human IgG antibody (Southern Biotech, Birmingham, AL) at 1:2,000 dilution with PBST-1% BSA. After washing, color was developed using *para*-nitrophenyl phosphate (*p*-NPP) and the absorbance was measured using 405 nm detection and 630 nm reference wavelength. The assay was performed twice per month or weekly in a batch of one or two ELISA plates at the Immunological Laboratory, Department of Immunology, Faculty of Medicine Siriraj Hospital, Mahidol University, Thailand. The relative concentration of anti-IFN-γ auto-Abs was described as the optical density (OD) of the patient’s sample compared to that of a healthy control, and an OD of at least 1 was considered a positive result [14, 15]. HLA genotyping was done with the next generation sequencing platform using GS Junior System (Roche/454 Life science, Branford, USA). High-resolution HLA genotypes were called with Connexio Assign ATF 454 software v1.1.0.33 (Connexio Genomics, a subsidiary of Illumina, San Diego, USA) as previously described [15]. Mycobacterial culture and identification were performed using standard recommended procedures. *Mycobacterium abscessus* was not further classified into subgroups in this study. Disseminated infection was defined as infection of the same pathogen at more than 1 noncontiguous body site or isolation of the causative pathogen from blood or bone marrow.

### Statistical analysis

Data were analyzed using the Statistical Package for the Social Sciences (SPSS) for Windows (Version 18.0; SPSS, Inc., Chicago, IL, USA). Categorical data are presented as numbers or numbers and percentages, and continuous data are presented as medians and ranges or the interquartile ranges (IQRs). Laboratory parameters, including total white blood cell count (WCC), hematocrit (Hct), CRP, ESR, and anti-IFN-γ auto-Abs concentration, were compared between active and stable disease using the Mann-Whitney U test. Multiple binary logistic regression was performed to evaluate the independent factors associated with disease activity. Percentage accuracy in classification (PAC), which reflects the percentage of cases in which the disease activity was correctly classified, was used to determine an arbitrary cut-off point for converting a continuous variable into a categorical variable. The Friedman test was used to evaluate changes in anti-IFN-γ auto-Abs over time. A *p*-value less than 0.05 was regarded as statistically significant.

## Results

### Patient characteristics and opportunistic infections

A total of 80 patients with anti-IFN-γ auto-Abs were identified during the study period. The median age at baseline was 49.5 years (range: 20–76 years), and half of patients were female. Comorbid diseases were found in only 5 (6.3%) patients (type 2 diabetes mellitus in 3 and autoimmune disease in 2). Common clinical presentations included generalized (80%) or localized (13.8%) lymphadenopathy, prolonged fever (63.8%), and weight loss (48.8%). Hepatomegaly and splenomegaly were found in 25% and 13.8% of patients, respectively. Reactive skin diseases were observed in 46 (57.5%) patients (17 had Sweet syndrome, 11 had AGEP, 6 had EN, and 12 had a combination of different skin lesions). ANA was detected in 46 of 74 (62.2%) patients. The baseline characteristics of the 80 patients with anti-IFN-γ auto-Abs are shown in Table 1.

**Table 1:** Baseline characteristics of 80 patients with anti-interferon-γ autoantibodies.

	n (%)
Age (years), median (IQR)	49.5 (20–76)
Male gender	39 (48.8)
Thailand region of birth	
Central	24 (30.0)
Northeast	21 (26.2)
North	18 (22.5)
West	10 (12.5)
South	7 (8.8)
Comorbid diseases	5 (6.3)
Reactive skin diseases	46 (57.5)
First proven opportunistic infection (OI) (n = 79)	91
All NTM species	48 (52.7)
*M*. *abscessus*	33 (36.2)
*M*. *fortuitum*	7 (7.7)
*M*. *avium* complex (MAC)	3 (3.3)
*M*. *scrofulaceum*	2 (2.2)
Other NTM spp.	3 (3.3)
*Salmonella* spp.	18 (19.8)
*M*. *tuberculosis*	12 (13.2)
Fungal infection^ϯ^	6 (6.6)
VZV	6 (6.6)
*Burkholderia pseudomallei*	1(1.1)
HLA studies (n = 68)	
HLA-DRB1	
DRB1*15	44 (64.7)
DRB1*15:01	18 (26.5)
DRB1*15:02	26 (38.2)
DRB1*16	48 (70.6)
DRB1*16:01	4 (5.9)
DRB1*16:02	39 (57.4)
DRB1*16:09	5 (7.3)
HLA-DQB1	
DQB1*05	
DQB1*05:01	38 (58.5)
DQB1*05:02	48 (70.6)
DQB1*05:03	1 (1.5)

^ϯ^Cryptococcosis 3 cases, talaromycosis 2 cases, histoplasmosis 1 case

Abbreviations: IQR, interquartile range; OI, opportunistic infection; NTM, nontuberculous mycobacteria; VZV, Varicella zoster virus; HLA, human leukocyte antigen

**Fig 1:**
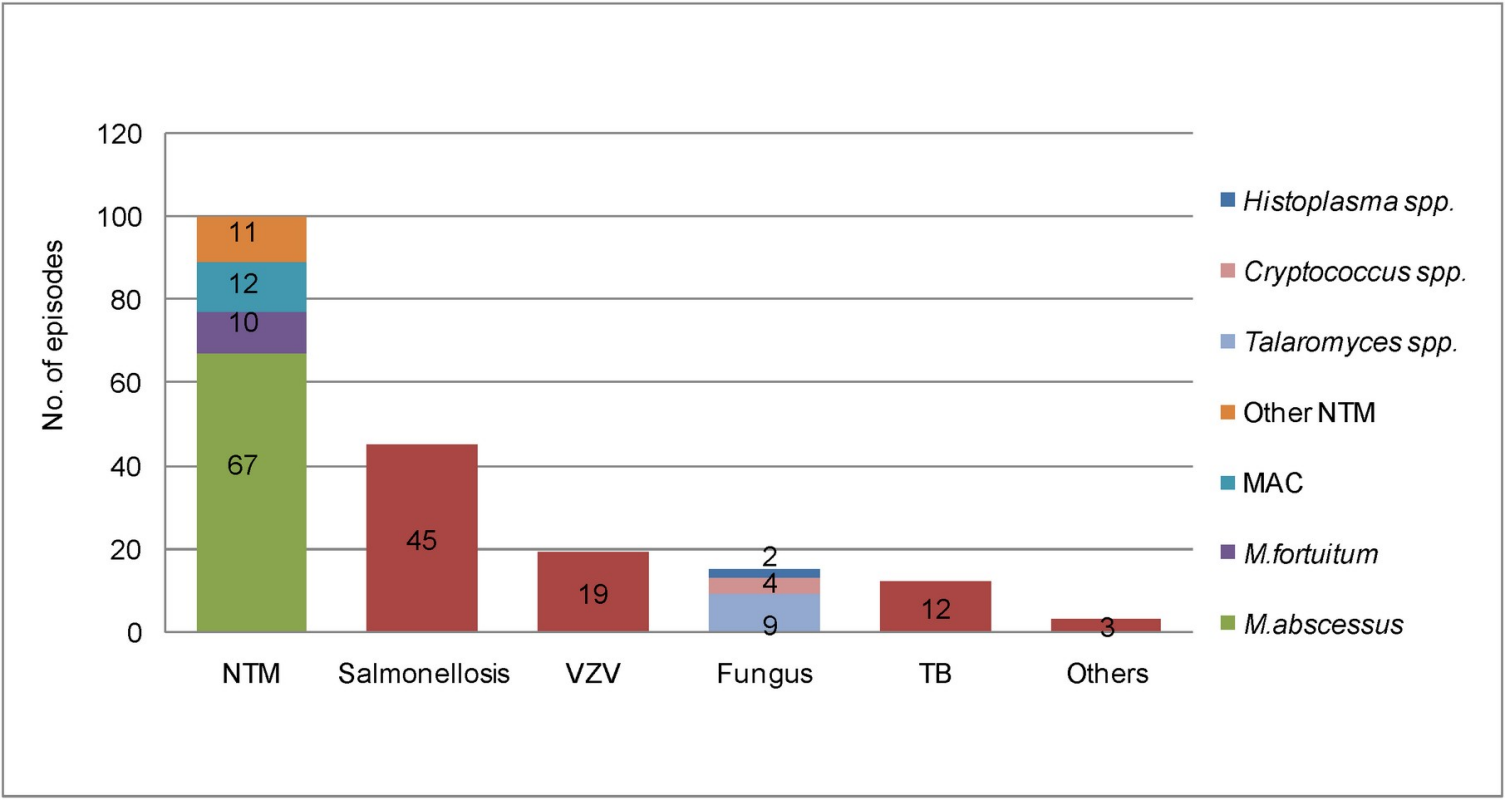
Distribution of 194 proven opportunistic pathogens diagnosed during the study period. (Abbreviations: NTM, nontuberculous mycobacteria; VZV, Varicella zoster virus; TB, tuberculosis; MAC: *Mycobacterium avium* complex).

We identified at least one OI in all patients except one. Although this patient presented with prolonged fever, weight loss, and multiple suppurative skin lesions, bacterial and mycobacterial culture as well as molecular identification of bacteria from various specimens, including blood, pus, and skin biopsy, detected no pathogen in this patient. He slowly deteriorated during empiric antibiotic and antimycobacterial therapy, but his clinical signs were resolved 2 weeks after plasma exchange. After being discharged from the hospital, this patient was lost to follow-up. The other 79 patients had 194 proven OIs during the study period (Fig 1), with a median of 2 OIs (range: 1–11). Ten (12.7%) patients presented with multiple OIs at the first episode (1 patient had 4 OIs, and 9 patients had 2 OIs). Subsequent OIs occurred in 48 (60.8%) patients, with a median duration between OI episodes of 8.3 months (range: 1.1–171.5). *Mycobacterium abscessus* was the most commonly identified OI [67 (34.5%) episodes], followed by *Salmonella* spp. [45 (23.2%) episodes] and other NTM spp. [33 (17%) episodes]. Overall, 100 episodes of NTM infection occurred in 65 patients. *M*. *abscessus* was identified predominantly in lymph nodes (38/67 episodes: 56.7%) and in blood culture (19/67 episodes: 28.3%), whereas *Salmonella* spp. was identified mainly in blood (32/45 episodes: 71.1%).

### Treatment outcome

**Fig 2:**
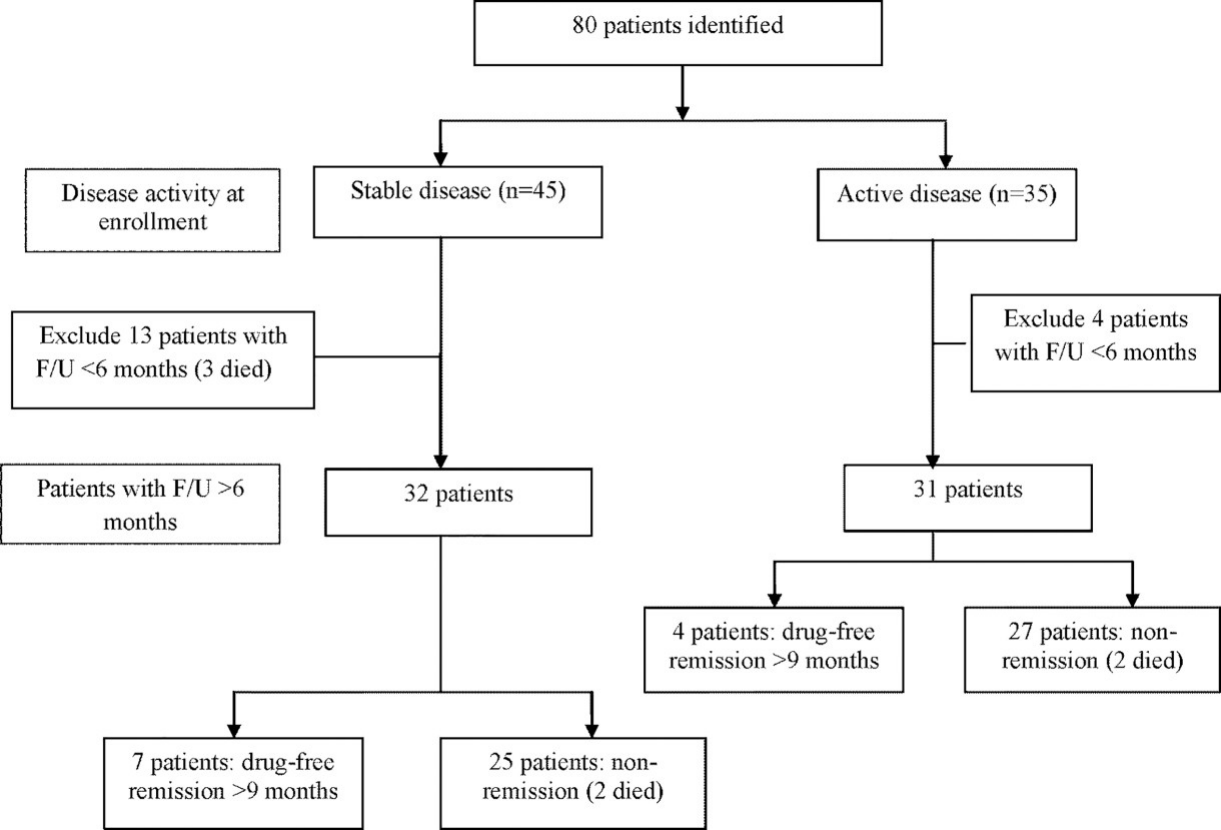
Flow diagram describing the overall clinical course of 80 patients with detectable anti-IFN-γ auto-Abs.

A flow diagram that describes the clinical course and outcome of all patients is shown in Fig 2. Overall, we excluded 17 patients from the long-term outcome analysis (13 patients in the group with stable disease and 4 patients in those with active disease at baseline) because they were either lost to follow up or they were followed up for less than 6 months. Among those who were included in the analysis, 34 (53.9%) patients had NTM disease at their initial clinical presentation. The median duration of NTM disease treatment was 27.4 months (range: 3.4–79.4 months). A combination of two parenteral antibiotics (amikacin and/or imipenem and/or cefoxitin) was given for 2 to 4 weeks, followed by a combination of oral macrolides (azithromycin or clarithromycin) and oral quinolones (levofloxacin or moxifloxacin or ciprofloxacin) for maintenance treatment. Other initial OIs included nontyphoidal salmonella infection (14 patients), VZV infection (5 patients), tuberculosis (7 patients), and others (3 patients); this group received antimicrobial treatment for a median duration of 16.2 months (range: 0.7–68.8). Twenty-eight of 44 (63.6%) patients developed relapse or recurrence of symptoms and/or signs (e.g., enlarged lymph node or reactive skin disease) after antimicrobial therapy for their initial OI was discontinued. The median drug-free interval between the first and second course of treatment was 8.8 months (range: 0.5–56.5 months).

Overall, 11 (17.5%) patients were classified as being in the drug-free remission, whereas 52 (82.5%) were classified as experiencing non-remission (including 4 patients who died during the study period). The median duration of follow-up in patients with drug-free remission and those with non-remission was 3.5 years (range: 1–4.4 years) and 2.5 years (range: 0.6–4.8 years), respectively. Causes of death were uncontrolled disseminated OI in two patients (disseminated *M*. *abscessus* infection in 1 and disseminated microsporidial infection in another) and suspected disseminated NTM in 2 patients who died at home. The median time to death after diagnosis of the initial OI was 15.6 months (range: 9.1–27.5 months). Comparison of baseline characteristics between the drug-free remission and non-remission groups is shown in Table 2. The baseline ESR and OD of anti-IFN-γ auto-Abs were both significantly different between the two groups.

**Table 2:** Baseline characteristics compared between the drug-free remission group and the non-remission group.

Characteristics	Drug-free remission	Non-remission	*p*-value
	(n = 11)	(n = 52)	
	n (%) or median (IQR)	n (%) or median (IQR)	
Male gender	7 (63.6)	23 (44.2)	0.242
Age (years)	49 (24–73)	50 (30–68)	0.640
Initial OI			0.331
NTM disease	8 (72.7)	26 (50.0)	
Salmonella spp.	1 (9.1)	13 (25.0)	
TB	1 (9.1)	6 (11.5)	
VZV infection	1 (9.1)	4 (7.7)	
Others	0	3 (5.8)	
Reactive skin lesion	6 (54.5)	31 (59.6)	0.756
Number of total OIs			
One	5 (45.5)	16 (30.8)	0.348
More than one	6 (54.5)	36 (69.2)	
Laboratory parameters			
Hematocrit (%)	31.4 (20.6–44.6)	29.9 (13.5–43.4)	0.388
White cell count (cells/μL)	15,490 (7,500–56,580)	20,740 (6,130–46,360)	0.707
ESR (mm/h)	21.0 (6.0–108.0)	84.0 (11–123)	0.002
CRP (mg/L)	11.1 (0.6–94.8)	53.5 (0.55–311.3)	0.093
Albumin (g/dL)	3.3 (2.6–4.7)	3.3 (1.5–4.3)	0.842
Globulin (g/dL)	5.7 (3.5–6.7)	5.4 (2.7–7.9)	0.567
OD of anti IFN-γ auto-Abs	3.14 (1.57–4.38)	3.86 (1.38–4.61)	0.018
HLA study	**(n = 11)**	**(n = 46)**	
HLA-DRB1-15:01	3 (27.3)	12 (26.1)	0.936
HLA-DRB1-15:02	3 (27.3)	19 (41.3)	0.390
HLA-DRB1-16:01	1 (9.1)	2 (4.3)	0.527
HLA-DRB1-16:02	5 (45.5)	30 (65.2)	0.226
HLA-DRB1-16:09	0	3 (6.5)	0.384
HLA-DQB1-05:01	7 (63.6)	25 (54.3)	0.577
HLA-DQB1-05:02	6 (54.5)	36 (78.3)	0.109
HLA-DQB1-05:03	1 (9.1)	0	0.039

A *p*-value<0.05 indicates statistical significance.

Abbreviations: IQR, interquartile range; OI, opportunistic infection; NTM, nontuberculous mycobacteria; TB, tuberculosis; VZV, Varicella zoster virus; ESR, erythrocyte sedimentation rate; CRP, C-reactive protein; OD, optical density; anti-IFN-γ auto-Abs; anti-interferon-γ autoantibodies; HLA, human leukocyte antigen

**Fig 3:**
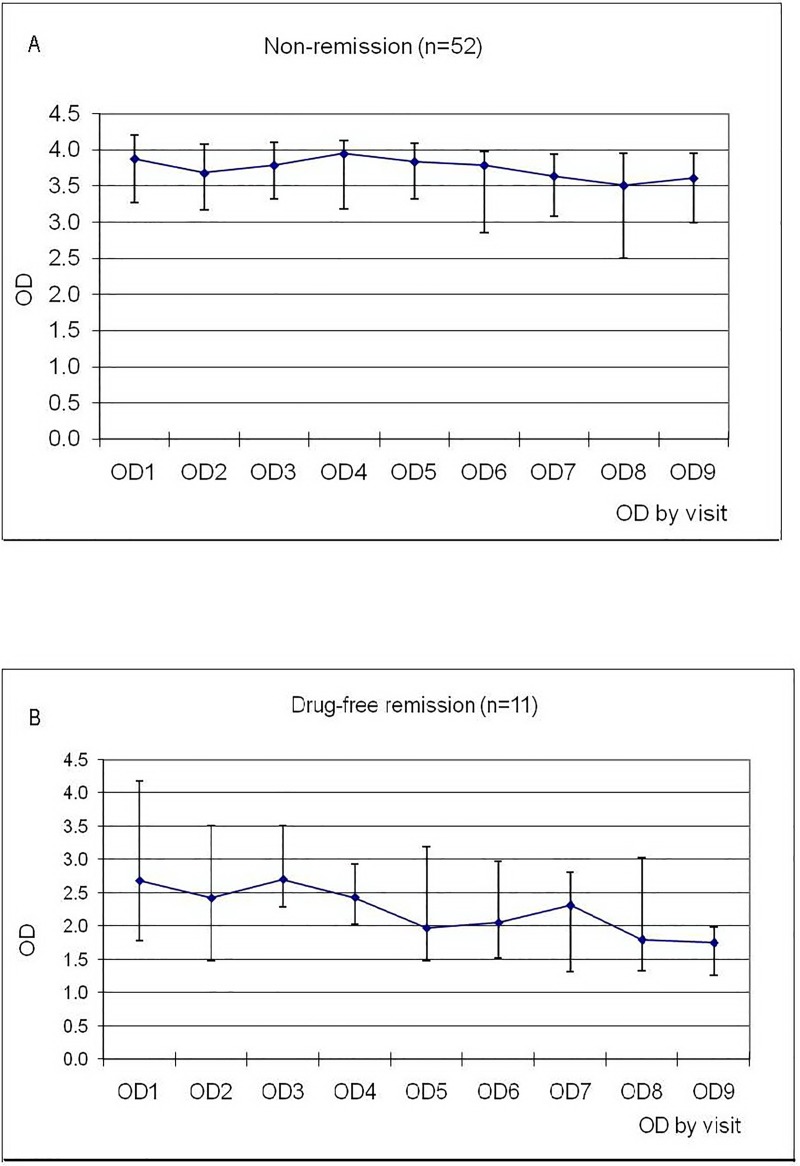
The distribution of optical density (OD) for anti-IFN-γ auto-Abs (median, interquartile range) taken every 3–6 months during the study period. (A) OD in the non-remission group.(B) OD in the drug-free remission group.

In the drug-free remission group, seven patients, three patients, and one patient received one, two, and three courses of antimicrobial therapy, respectively, with a median duration of illness prior to remission of 5.2 years (range: 1.9–9.7 years). The median duration of drug-free remission was 2.2 years (range: 0.9–4.6 years). The concentrations (OD) of anti-IFN-γ auto-Abs (median, IQR) taken every 3–6 months during the follow-up period in both groups are shown in Fig 3A and Fig 3B. The follow up OD was significantly decreasing over time only in the drug-free remission group (*p* = 0.001). Two patients in the drug-free remission group and one in the non-remission group had undetectable anti-IFN-γ auto-Abs during the study period. At the last sampling, the median concentration of anti-IFN-γ auto-Abs in the drug-free remission group was significantly lower than that in the non-remission group (1.29 [IQR: 1.26, 1.75] *vs*. 3.36 [IQR: 2.5, 3.9], respectively; *p*<0.001).

### Laboratory markers associated with disease activity

At baseline, 31 and 32 patients had active and stable disease status, respectively. Laboratory parameters found to be significantly different between the active and stable disease episodes included Hct, WCC, CRP, ESR, and anti-IFN-γ auto-Abs (Table 3). CRP showed the best performance for disease assessment, with an area under the receiver operating characteristic (ROC) curve (AUC) of 0.88 (95% CI: 0.81–0.95). The optimum cut-off value of CRP for accurately differentiating between active and stable disease episodes was 30 mg/dL. This cut-off had a maximum risk ratio of 3.63 (95% CI: 1.83–7.18) after being adjusted for ESR, WCC, Hct, anti-IFN-γ auto-Abs, and interaction between Hct and ESR (*p* = 0.95). The AUCs (95% CI) of ESR, WCC, Hct, and anti-IFN-γ auto-Abs were 0.81 (0.73–0.89), 0.80 (0.72–0.89), 0.71 (0.61–0.81), and 0.69 (0.60–0.79), respectively. The optimum cut-off values and performance of these laboratory parameters for differentiating between active and stable disease episodes are shown in Table 4.

**Table 3:** Laboratory parameters compared between active and stable episodes for 63 patients.

Variables	Active Median (IQR)	Stable Median (IQR)	*p*-value
Hematocrit	33.3 (29.6–39.4)	38.6 (35.4–42.1)	<0.001
WCC (×10^3^/μL)	14,560 (9,302–20,592)	7,890 (6,310–10,310)	<0.001
CRP (mg/L)	52.2 (30.6–90.3)	4.2 (2.0–8.4)	<0.001
ESR (mm/h)	80 (47–100)	31.5 (17.0–56.0)	<0.001
OD anti-IFN-γ	3.9 (3.5–4.1)	3.3 (2.6–3.9)	<0.001
auto-Abs			

A *p*-value<0.05 indicates statistical significance

Abbreviations: IQR, interquartile range; WCC, white cell count; CRP, C-reactive protein; ESR, erythrocyte sedimentation rate; OD, optical density; anti-IFN-γ auto-Abs; anti-interferon-γ autoantibodies

**Table 4:** Performance of laboratory parameters for determining active disease.

Variables	Cut-off values	Sensitivity (95% CI)	Specificity (95% CI)	Accuracy (95% CI)
CRP (mg/L)	≥30	77% (64%-87%)	93% (84%-97%)	86% (78%-91%)
WCC (×10^3^/μL)	≥11,000	71% (58%-82%)	85% (73%-92%)	78% (70%-85%)
ESR (mm/h)	≥42	79% (65%-88%)	67% (54%-78%)	72% (63%-80%)
Hematocrit (%)	<35	60% (46%-72%)	76% (64%-85%)	69% (59%-76%)
OD of anti-IFN-	≥3.5	77% (64%-86%)	58% (45%-69%)	67% (58%-75%)
γ auto-Abs				

Abbreviations: CI, confidence interval; CRP, C-reactive protein; WCC, white cell count; ESR, erythrocyte sedimentation rate; OD, optical density; anti-IFN-γ auto-Abs; anti-interferon-γ autoantibodies

## Discussion

Herein, we described the clinical presentation, clinical course and treatment outcome of 80 patients with detectable anti-IFN-γ auto-Abs diagnosed in a single hospital in Thailand. In all except one patient, initial clinical presentations were associated with disseminated OIs. Although the clinical presentation of that patient was very similar to that of the other patients, his condition deteriorated despite all available antimicrobial, including antimycobacterial therapies. His dramatic clinical improvement after plasma exchange therapy raises the possibility that immunoglobulin might have played a role in his autoimmunity or immunodeficiency. Unfortunately, we had no additional information regarding this patient after his discharge.

Disseminated NTM disease was confirmed as the most common OI associated with this clinical syndrome [12, 13]. However, the distribution of NTM species varied. *M*. *abscessus* was the most common NTM pathogen found in this study and in other case series from Thailand [3, 6, 9, 14], but *M*. *avium* complex (MAC) was the most common NTM pathogen reported from studies conducted in Taiwan and Japan [12, 13]. This may reflect geographic differences in the distribution of NTM in the general population. More epidemiological data on NTM diseases in Thailand are needed to prove this assumption. In addition to NTM diseases, other OIs, including nontyphoidal salmonella, TB, VZV, and fungal pathogens, especially *T*. *marneffei*, were identified as either the initial clinical manifestation, coinfection, or subsequent infection developing after the NTM disease episode. Based on our results and other published data [14], adult-onset immunodeficiency syndrome due to anti-IFN-γ auto-Abs should be suspected not only in patients with disseminated NTM diseases but also in otherwise healthy individuals who present with disseminated or recurrent infections with these opportunistic pathogens, as well as in patients presenting with reactive skin disease.

After diagnosis, these patients required prolonged duration of antimicrobial treatment or re-treatment and long-term follow-up. The clinical courses were consistent with both published case series from Taiwan and Japan [12, 13]. The duration of combined antimycobacterial therapy for patients with disseminated NTM diseases is approximately two years. However, the optimal treatment regimens and their duration for treating disseminated NTM diseases remain unclear.

To determine the factors associated with a better outcome in this population, we arbitrarily classified our patients into a drug-free remission group if we were able to discontinue antibiotic treatment for at least 9 months (because the median duration between the discontinuation of treatment and the next episode of OI was 8.8 months in our study) and a non-remission group if they did not meet the criteria. We continue to follow these patients to determine their longer term outcomes. This information would help to confirm or to determine a more rational criterion to classify the long term outcome than the 9-month treatment free period that we used in this study. Despite persistent detection of anti-IFN-γ auto-Abs, approximately 1 in 5 patients achieved the drug-free remission criterion. We continue to follow these patients to determine their longer term outcomes.

The correlation between the baseline concentration of anti-IFN-γ auto-Abs and treatment outcome was inconsistent in previous studies [13, 16]. In this study, the baseline concentration of anti-IFN-γ auto-Abs was significantly lower in the drug-free remission group than in the non-remission group which was consistent with the data published from Taiwan [13]. However, the autoantibody level and neutralizing capacity were not associated with the clinical course or severity of disease among the three patients reported by Tham and colleagues [16]. In our study, we also observed that the concentration of anti-IFN-γ auto-Abs decreased significantly over time only in the drug-free remission group. Although the reduction in anti-IFN-γ auto-Abs levels can be either the cause or result of clinical remission, we postulate that it was more likely to be the cause. We did not observe a change in anti-IFN-γ auto-Abs during the stable disease state in the non-remission group. Consistent with previous study [15, 17], we only found high prevalence rates of 2 HLA class II subtypes; DRB1*15/16 and DQB1*05 among our patients with no significant differences in allele frequency of other HLA subtypes. To date, there is no enough data to make a conclusion regarding which alleles is more significantly associated with anti-IFN-γ auto-Abs. Although we found a higher prevalence of DRB1*15:02 DRB1*16:02 and DQB1*05:02 in non-remission group and DQB1*05:01 in remission group, there were no statistically significance.

There is currently no treatment that effectively reduces the concentration of anti-IFN-γ auto-Abs in this population. Anti CD-20 therapy or rituximab has been successfully applied as an adjunct along with antimicrobial treatment in a small number of patients [18]. Therefore, antimicrobial therapy remains the mainstay treatment for this clinical syndrome. Disease activity was determined based on symptoms and signs at each follow-up visit, and patients with stable disease were either switched from parenteral to oral antibiotic treatment or continued or discontinued oral antibiotic therapy. Patients with active disease were either restarted or continued antibiotic therapy. We also observed a significant bone marrow response in this population [9, 13], whereby they were significantly more anemic and leukocytosis during the active disease episode than during the stable disease episode.

Various inflammatory biomarkers, such as ESR and CRP, are commonly used in clinical practice to supplement clinical data for determining disease activity in various autoimmune diseases and chronic infectious diseases [19, 20]. We confirmed that both ESR and CRP were also increased during active disease episodes, similar to the response of these parameters in other autoimmune diseases and unresolved bacterial infections, such as osteomyelitis [21]. Such a finding was also mentioned in a published series from Taiwan [13]. In addition, the cut-off value that yielded the best performance for differentiating stable from active disease for each parameter was determined in this study were as follows: CRP of 30 mg/L, ESR of 42 mm/h, WCC of 11,000/mm^3^, hematocrit of 35%, and OD of anti-IFN-γ auto-Abs of 3.5. More standardized and simple method to compare the activity of anti-IFN-γ auto-Abs than OD is under evaluation in Thailand. At the moment the OD /or combination of these laboratory parameters may be useful for determining disease activity and the duration of antimicrobial treatment.

In summary, data from this largest cohort study ever reported confirmed that reinfection or relapse of OIs, especially NTM infections, is common among patients with detectable anti-IFN-γ auto-Abs, despite a long duration of antimicrobial treatment. ESR and CRP may be useful as an adjunct to the clinical assessment of disease activity during follow-up. Baseline and changes in anti-IFN-γ auto-Abs levels were found to significantly correlate with the treatment outcome. Treatment to modify anti-IFN-γ auto-Abs production may improve long-term outcomes in these patients.
